# Uncovering Distinct Drivers of Covert Attention in Complex Environments With Pupillometry

**DOI:** 10.1111/psyp.70036

**Published:** 2025-03-19

**Authors:** Yuqing Cai, Stefan Van der Stigchel, Julia Ganama, Marnix Naber, Christoph Strauch

**Affiliations:** ^1^ Experimental Psychology, Helmholtz Institute Utrecht University Utrecht the Netherlands

**Keywords:** complex environment, covert attention, pupillometry

## Abstract

Spatial visual attention prioritizes specific locations while disregarding others. The location of spatial attention can be deployed without overt movements (covertly). Spatial dynamics of covert attention are exceptionally difficult to measure due to the hidden nature of covert attention. One way to implicitly index covert attention is via the pupillary light response (PLR), as the strength of PLR is modulated by where attention is allocated. However, this method has so far necessitated simplistic stimuli and targeted only one driver of covert attention per experiment. Here we report a novel pupillometric method that allows tracking multiple effects on covert attention with highly complex stimuli. Participants watched movie clips while either passively viewing or top‐down shifting covert attention to targets on the left, right, or both sides of the visual field. Using a recent toolbox (Open‐DPSM), we evaluated whether luminance changes in regions presumably receiving more attention contribute more strongly to the pupillary responses—and thereby reveal covert attention. Three established effects of covert attention on pupil responses were found: (1) a bottom‐up effect suggesting more attention drawn to more dynamic regions, (2) a top‐down effect suggesting more attention towards the instructed direction, and (3) an overall tendency to attend to the left side (i.e. pseudoneglect). Beyond the successful validation of our method, these drivers of covert attention did not modulate each other's effects, indicating independent contributions of bottom‐up, top‐down, and pseudoneglect to covert attention in stimuli as dynamic as the present. We further explain how to use Open‐DPSM to track covert attention in a brief tutorial.

## Impact Statement

Previous studies have shown that the pupillary light response (PLR) can physiologically index covert attention, but only with highly simplistic stimuli. With a newly introduced technique that models the contribution of luminance changes across the visual field to pupil size changes, we demonstrated for the first time that the PLR can index distinct drivers of covert attention (bottom‐up, top‐down, and pseudoneglect effects) simultaneously even in dynamic and complex environments.

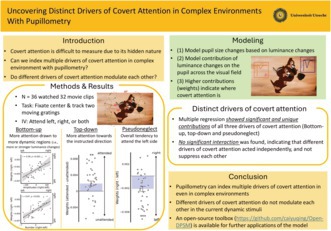

## 1 Introduction

The rich visual input reaching our eyes exceeds our transmission and processing capacities. As a consequence, spatial attention, a selective mechanism that amplifies relevant locations and disregards irrelevant ones, is indispensable for the effective processing of visual input (Posner et al. 1980). Visuospatial attention can be allocated to a given location either by shifting the gaze to it (overt attention) or by maintaining the gaze elsewhere but attending to it with one's peripheral vision (covert attention; i.e. attending to a location without moving the eyes) (James 1890; von Helmholtz 1867). Covert attention improves the perception of stimuli and facilitates the monitoring of the surroundings (Carrasco 2011, 2018; Nakayama and Mackeben 1989; von Helmholtz 1867) without the need for cognitively more costly saccades (Koevoet et al. 2023), enabling efficient responses to environmental changes. For instance, a driver can fixate on the road straight ahead while simultaneously being aware of peripheral hazards. When a motorcycle suddenly approaches the vehicle from the periphery, this may draw covert attention, ensuring that the driver can react promptly. In this case, the abrupt onset of a salient stimulus (the motorcycle) drives the shift of covert attention in a bottom‐up manner, which is commonly known as exogenous attention (Barbot et al. 2011; Carrasco et al. 2002; Pestilli and Carrasco 2005; Phelps et al. 2006; Prinzmetal et al. 2005; Yantis and Jonides 1990). Alternatively, if the driver is expecting approaching traffic from the right, the driver is likely to voluntarily shift their covert attention to the right in anticipation of a potentially relevant stimulus. This top‐down modulation of attention towards a designated spatial location is commonly referred to as endogenous attention (Giordano et al. 2009; Hikosaka et al. 1993; Mangun and Hillyard 1990; Sperling and Melchner 1978; Yeshurun et al. 2008). In addition, the attention of the driver may be inherently biased, as young adults exhibit an overall subtle leftward bias in spatial covert attention, observable in various tasks and measurement methods (e.g. Jewell and McCourt (2000); Mattingley et al. (2004); Strauch et al. (2022a)).

Due to its importance for visual perception, the effects of covert attention are studied extensively. Perceived visual properties (e.g. contrast sensitivity, spatial resolution or brightness) of the attended stimuli are boosted, and they are responded to more accurately and quickly (Cameron et al. (2002); Carrasco et al. (2004); Posner et al. (1980); von Helmholtz (1867); Yeshurun et al. (2008); see Carrasco (2011) for a review). While the allocation of overt attention can be studied relatively easily using eye trackers, the allocation of covert attention is harder to study due to its inherently hidden nature. The allocation of covert attention is therefore typically studied by measuring its consequences that is, by enhanced task performance at attended over unattended locations. For instance, in the Posner cueing paradigm, a location is first cued exogenously or endogenously, and a target subsequently appears either in the cued (valid) or uncued (invalid) location. Faster or more accurate responses to valid targets indicate the allocation of covert attention (e.g. Posner (1978); Posner et al. (1980); Posner and Cohen (1984)).

As alternatives to these behavior‐based measures, physiological measures of covert attention have been adopted. For instance, microsaccade directions can inform about the direction of covert attention relative to fixation (e.g. Engbert and Kliegl (2003); Hafed and Clark (2002); Liu et al. (2022); van Ede (2023) but see Willett and Mayo (2023)), and the lateralization of alpha oscillations in EEG/MEG signals can uncover the attended hemisfield (e.g. Gould et al. (2011); Ikkai et al. (2016)). While spatially coarse, such physiological signals can uncover the otherwise imperceptible allocation of covert attention without the need for explicit responses. We here focus on another physiological measure that has the potential to be particularly powerful to this end, namely attentional modulations of the pupillary light response (PLR).

Pupillary light responses refer to the pupil dilating when brightness decreases and constricting when brightness increases. The PLR regulates the amount of light entering the eye and balances visual sensitivity and acuity (Campbell and Gregory 1960; Mathôt and Van der Stigchel 2015; Woodhouse and Campbell 1975). However, the PLR is not merely a reflexive low‐level response to luminance, as the conventional point of view assumed (Loewenfeld 1958). Studies have shown that attention to stimuli with different brightness, in the external world or the mind, modulates the PLR, be it in visual awareness (Harms 1937; Naber et al. 2011), subjective interpretation or imagination (Binda et al. 2013b; Castellotti et al. 2020; Naber and Nakayama 2013; Piltz 1899) and covert attention (Binda et al. (2013a); Haab (1886); Naber et al. (2013); see Strauch (2024) for a historical review). Hereby, covert attention to bright locations induces pupil constriction, whereas covert attention to dark locations induces dilation (Binda et al. 2013a; Mathôt et al. 2013). Similarly, covert attention amplifies pupil light and dark responses to luminance change (Naber et al. 2013). In other words, covert attention to one location will modulate pupil size changes to the luminance change in this location, in the same direction as the effect of corresponding physical luminance change on pupil responses. For example, Mathôt et al. (2013) demonstrated that the PLR is modulated by the luminance changes of the attended location (*d* > 1), and this index of covert attention also predicted behavioral detection performance, as targets were better detected when the attentional modulation of the PLR was stronger (*r* = 0.45–0.59). By now, this mechanism is frequently exploited to physiologically uncover covert attention across a range of domains and tasks (Binda and Gamlin 2017; Fabius et al. 2017; Mathôt et al. 2013, 2016; Salvaggio et al. 2022; Strauch et al. 2022b; Ten Brink et al. 2023; Wagenvoort et al. 2020). Together, these findings suggest that the PLR can physiologically, unobtrusively, and continuously index the location of covert attention. As both pupil responses to luminance increments and luminance decrements are affected by covert attention (Binda et al. 2013a), we here use the term PLR to denote pupillary responses to both dark and bright stimuli.

Still, the PLR can thus far only be used to index covert attention with simplistic stimuli, such as black and white patches (Binda et al. 2013a) or backgrounds (Mathôt et al. 2013), again limiting how covert attention can be studied. This stimulus control is necessary due to the various factors affecting pupil size as measured by video‐based eye trackers (see Mathôt (2018); Strauch et al. (2022b) for reviews). In other words, although the PLR is a less obtrusive physiological index of covert attention than behavioral measures in that it requires no manual responses, the stimuli that can be used are severely limited. Referring back to the driving example, it would not be possible to infer where covert attention is directed using the PLR due to the complexity of the stimulus material.

Here, we introduce and evaluate a novel method to measure covert attention with the PLR using complex and dynamic stimulus material (movies). Participants fixated at the center whilst movies were played, and they were either instructed to direct covert attention to the left, right, both sides, or just passively view. Our approach allows us to investigate three distinct effects on covert attention in parallel: (1) bottom‐up effect predicting that the more salient side of the movie would attract more attention. Here, saliency was approximated by the number and amplitude of luminance changes that the side with more frequent and stronger luminance changes was considered more salient; (2) top‐down effect predicting that more attention should be allocated to the instructed to‐be‐attended side than the unattended side, and (3) pseudoneglect effect predicting that the left side would generally attract more attention. We intended to show that all three effects can be uncovered with the PLR. To this end, we modeled how strongly visual changes in the movies affected the PLR across the visual field, using our modeling toolbox (Open Dynamic Pupil Size Modeling [Open‐DPSM]; Cai et al. (2024)). The model estimates the relative contribution of visual changes to pupil responses across the visual field with observed pupil size changes. We demonstrate that the estimated contribution of visual events to the pupil responses at different locations is coherent with the allocation of covert attention, that is, the presumably attended locations affect pupil size more—and thereby show that the location of covert attention in complex stimuli can be inferred with pupillometry. This successful inference of covert attention further allows unique insights into covert attention. Previous studies have shown key differences among these effects on behavioral performances (Barbot et al. 2012; Yeshurun et al. 2008), which suggest likely separate processes. However, it is still debatable whether those drivers modulate each other, that is, whether one effect would be suppressed or attenuated by the presence of other effects based on the results of behavioral tasks (Berger et al. 2005; Berlucchi et al. 2000; Chica et al. 2013; Yarbus 1967). Hence, the current study also intended to capture all three drivers of covert attention concurrently with pupillometric measurements in dynamic scenes to assess whether those effects are modulated by the others.

## 2 Methods

### 2.1 Participants

Forty healthy participants (*M*_age_ = 24.3, SD_age_ = 2.9 years; 32 women, 8 men) took part in the experiment. All participants had normal or corrected‐to‐normal vision. The study was approved in advance by the local ethics board of the Faculty of Social Sciences of Utrecht University (22‐530).

### 2.2 Apparatus

The stimuli were displayed on a large OLED 65B8PLA LG 65' TV (145 by 80 cm; 88.1° by 56.1° visual angle) at a resolution of 1920 by 1080 pixels and a refresh rate of 60 Hz.

Participants sat 75 cm away from the screen and their movement was restricted by a chin‐ and forehead‐rest. Eye movement and pupil size data were recorded binocularly with a tower‐mounted Eyelink 1000 (SR Research) at 500 Hz. The experiment was implemented with the Python‐based Psychopy package to present stimuli, record responses, and synchronize events with the Eyelink (version 2022.2.4, Peirce et al. 2019). The experiment was conducted in a dark room with the Eyelink monitor as the only source of light except for the TV that displayed the stimuli.

### 2.3 Stimuli and Procedure

Thirty‐two 60 s movie clips were presented to participants (selected from a previously used database, see https://doi.org/10.34894/LEYVL8). Of these, 18 were cartoon/animated movies and 14 were live‐action movies. As the screen was larger than the eye‐tracker's scope, the movies were presented centrally with a size of 1152 by 648 pixels (60.2° by 35.5° visual angle), which occupied 60% of the screen. The remaining screen area was covered by a black background, consistent with the luminance levels of the surrounding black wall behind the screen. Luminance changes in the movie were expected to evoke PLRs; the allocation of covert attention should consequently be illustrated by different influences of luminance changes on PLRs across the visual field (see below for further details).

As movies are rarely symmetric in visual input, this asymmetry was expected to induce bottom‐up effects, causing sides with more events to attract more attention and stronger effects of luminance changes on pupil responses. To control for the bottom‐up effects in investigating the top‐down effect (see below), movies were mirrored for 50% of participants.

**FIGURE 1:**
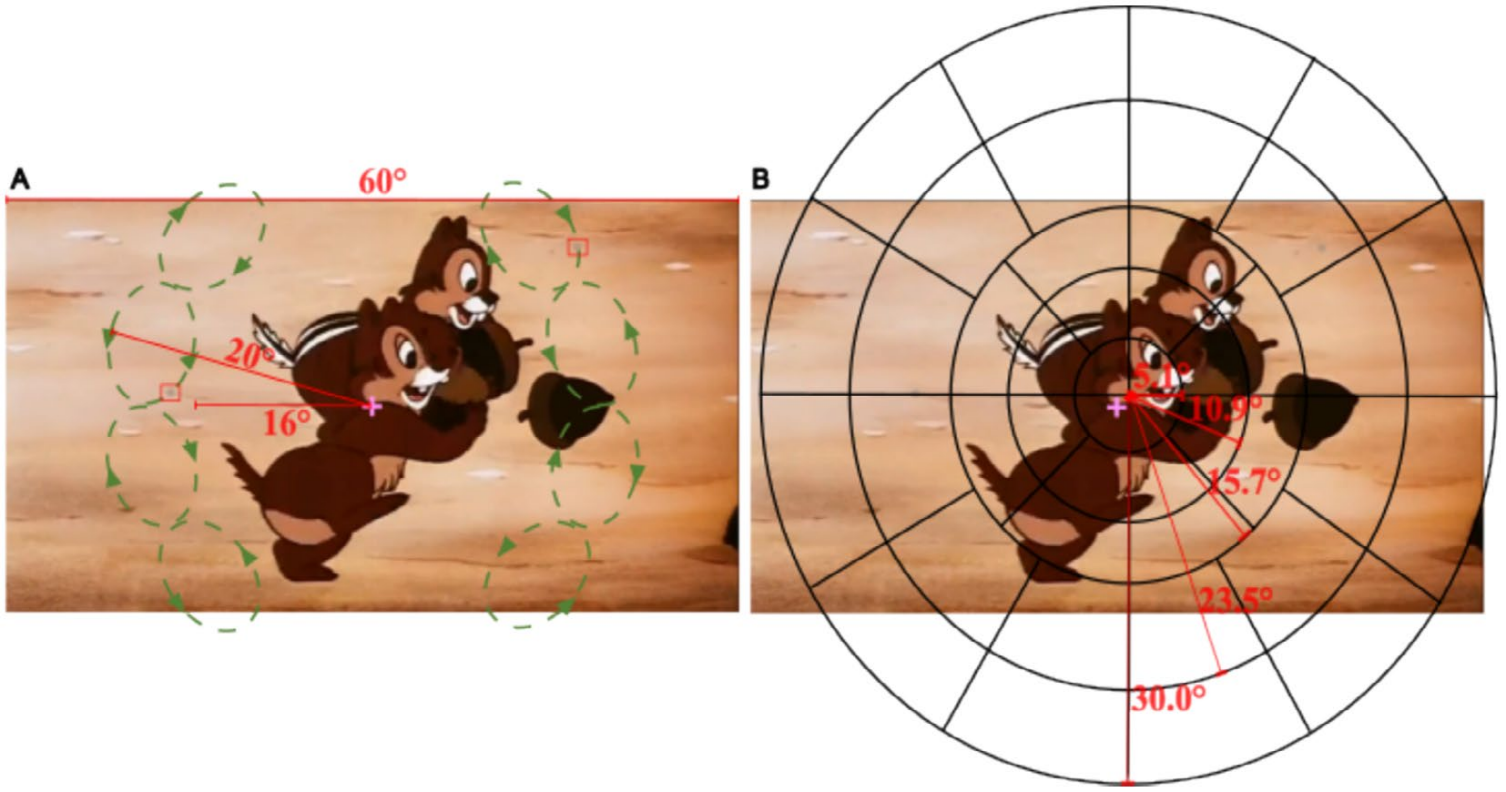
Experimental design. (A) Screenshot of a movie with gratings (in the red boxes) and a fixation cross. Overlaid green dashed lines with arrows denote the motion trajectories for the moving gratings; (B) the visual field was mapped into regions with five eccentricities (marked with black lines). In this example, the map center is marginally shifted towards the upper‐right quadrant of the movie, illustrating the gaze‐contingent modeling procedure.

A fixation cross was overlaid on the center of the movie (xyY 0.3209, 0.1542, 0.2848 in CIELAB space, 1.73° by 1.73° visual angle). To direct covert attention top‐down, two gratings generated by the Psychopy visual “RadialStim” function (opacity = 0.4, diameter = 1.13 cm, 0.86° by 0.86°) were moving in “double eight”‐shaped paths on the left and right of the visual field (see Figure 1 for a visualization of the trajectories). The gratings moved at a velocity of 7.00°/s (9.2 cm/s), starting from random points on the path for each movie. Gratings stopped randomly for 2–8 times at random time points for 1 s each in each movie, with at least one stop on either side. The stopping intervals of the two gratings were mutually exclusive, with a minimum interception of 3 s. The gratings' movements and pauses for each movie were predetermined to guarantee identical stimuli for all participants across all conditions (see https://osf.io/m5h2f/ for examples of the stimuli).

Participants fixated on the central cross throughout. In three out of four conditions, participants needed to indicate stops in to‐be‐attended gratings by pressing the space bar. To manipulate the allocation of covert attention, each participant was tested in four conditions: two conditions where participants responded to only left or right stops (“left” and “right” conditions); and two control conditions, one in which participants responded to stops on both sides (“both” condition); and one passive‐viewing condition without the need to report stops (“none” condition). Each condition featured eight trials (a trial being one movie with randomly occurring grating stops), presented in a random sequence per participant. Participants were informed of the condition before each trial and cued throughout the movie by a slightly modified fixation cross for the non‐passive conditions (thick leg to left for “left”, thick leg to right for “right”, both thick legs for left and right for “both”). Participants had to respond after each stop within 1.5 s and were instructed to prioritize accuracy over speed. Upon completion of each movie, performance was given as the ratio of correct responses (hits) among all possible responses (hits, misses, false alarms). If participants' gaze deviated more than 20% of the time by 1.44° (50 pixels) or more in a trial, they were warned, and the trial was repeated. Only the final finished trial for each movie was retained in the dataset.

The eye tracker was calibrated using a five‐point procedure at the start of the experiment and after every four trials. In addition, if a participant moved their head, a recalibration was forced, and the unfinished trial was repeated. Participants started the trials self‐paced by pressing “b” on the keyboard. To control for handedness, participants switched hands every four trials.

### 2.4 Data Analysis

#### 2.4.1 Preprocessing of Eye‐Tracking Data

Pupil change velocities exceeding three standard deviations were marked as blinks, removed, and interpolated. Eye‐tracking data were down‐sampled to match the sampling rate of the movie (25 Hz). As our model only accounts for pupil size changes, not absolute pupil size, the pupil data were *z*‐scored to standardize pupil size changes across trials and to simplify the analysis.

Despite controlled gaze positions during the experiment, eye movements were inevitable and occasionally occurred. To further mitigate the influence of the gaze, gaze positions that deviated more than 2.5° from the center were removed, together with 2 s after those time points.

There were no differences in the amount of missing data across conditions, both after removing blinks (*F*(3,140) = 1.23, *p* = 0.30) and after removing saccades (*F*(3,140) = 1.50, *p* = 0.22). Trials with more than 30% missing data post‐blink removal or 50% post‐gaze removal were excluded. After excluding invalid trials, each trial had 12.95% missing data on average.

Four participants were excluded: one due to unreliable eye‐tracking data caused by hanging eyelids, one due to calibration issues, one due to the exclusion criterion of ADHD (the diagnoses of the participants were only collected after the experiment), and one due to insufficient trials (fewer than two trials in at least one condition). The dataset obtained from the final 36 participants included equal number of participants watching mirrored and non‐mirrored movies.

#### 2.4.2 Modeling Pupil Size Changes

**FIGURE 2:**
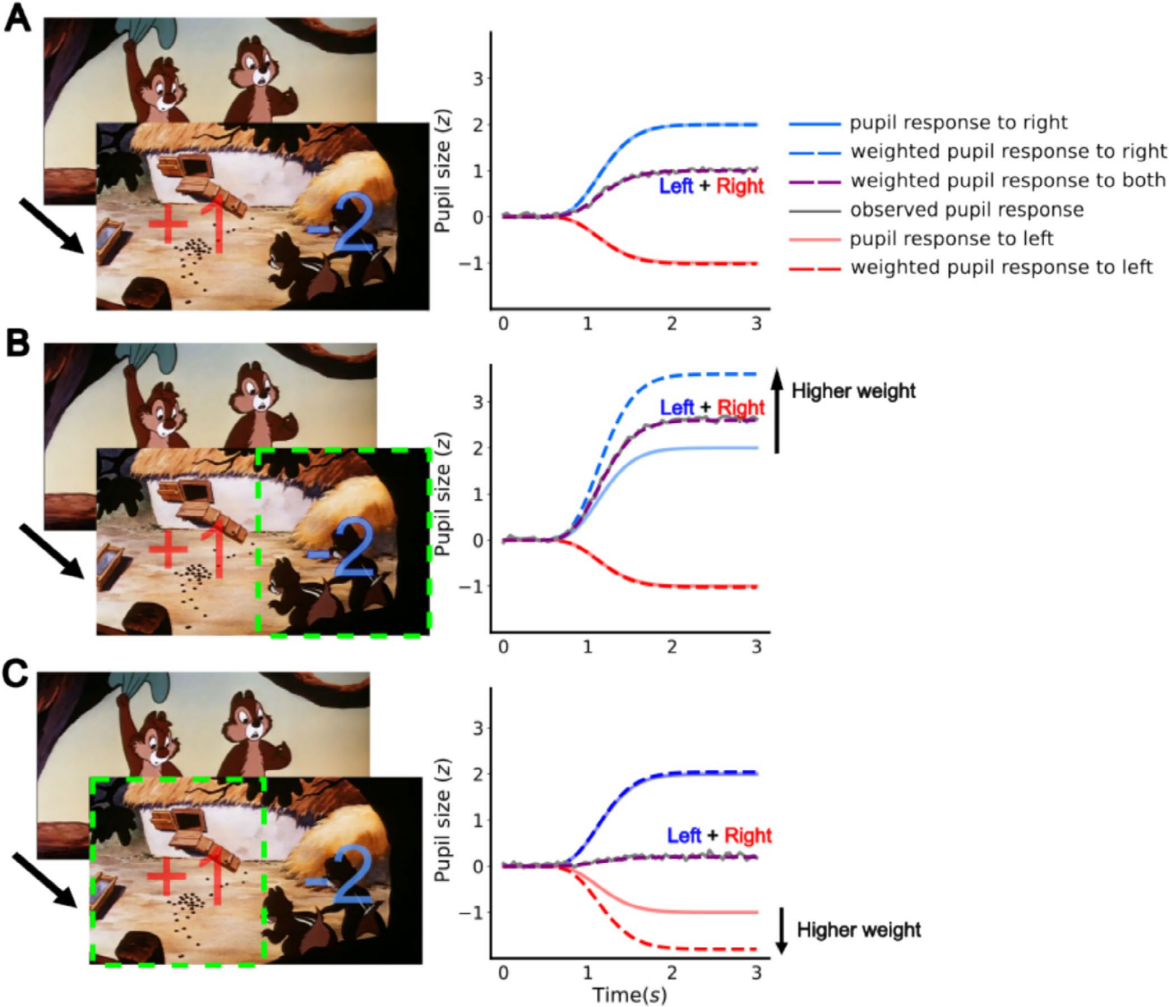
Interpretation of regional weights. A simplified illustration of how regional weights (reflecting the degree of attention) were obtained when modeling pupil size changes for three different attention shifts. (A) Attention equally allocated to left and right, (B) attended left, and (C) attended right. The left column displays the luminance changes between two consecutive movie frames for the two regions, with an increment of 1 in the left region and a decrement of 2 in the right region. Dashed green boxes indicate the attended regions. In the right column, solid blue and red lines depict the raw pupil responses to luminance changes in each region, regardless of attention (i.e. regional weights). Dashed blue and red lines represent the weighted pupil responses where attention increases the weights assigned to the luminance change in either the left or right image region. The overall pupil response (dashed purple line) combines the weighted responses from both regions. In Open‐DPSM, data inputs consist of observed pupil size changes (gray lines) and luminance changes, whereas regional weights are unknown. The model determines the best combination of regional weights such that the predicted pupil response (dashed purple lines) can be aligned optimally with the observed pupil response.

To obtain the relative strength of the effects of luminance changes on pupil responses across the visual field, an open‐source toolbox “Open‐DPSM” (Cai et al. 2024) was adopted to model pupil responses to luminance changes in the movie. Luminance changes were extracted per region gaze‐contingently for each frame to mimic the retinal image by re‐aligning the frame center with the gaze position (see Figure 1B for visual field regions and gaze‐contingent modeling procedure). Pupil size changes to luminance changes were modeled per region. The relative strength of pupil responses to luminance changes across the visual field are different. To account for these effects, regional weights were fitted to determine the relative contributions of luminance changes across regions to the overall pupil response. Consequently, regions with higher weights are regions that have a relatively stronger influence on the overall pupil response. All possible combinations of regional weights were tested until the model found an optimal combination that could best predict pupil size changes. For a straightforward description of regional weights, see https://github.com/caiyuqing/Open‐DPSM/ (“Regional weights illustration” folder) for an interactive demonstration of how the model obtains regional weights. It is important to note that the regional weights are different from the amplitude of pupil responses to luminance changes in each region. Rather, regional weights indicate the relative effects of luminance changes on pupil size changes among regions. For example, say there are two luminance changes with strengths 1 and −2 in two regions (see Figure 2), a luminance change with the strength of −2 would indeed elicit a stronger pupil response (whether dilation or constriction; Figure 2A), but this response is not directly related to the regional weights. In the absence of attentional effects, the amplitude of the pupil response to the luminance change with a strength of −2 would be double that of the response to the change with a strength of 1, resulting in an overall increase in pupil size (Figure 2A). As the pupil responded exactly according to the strength of the visual events in this example, the model would assign equal weights to both regions. Attention to any one of these two regions should let the respective luminance change have a stronger influence on the observed pupil size change than can be expected based on their physical amplitudes. The model thus assigns a higher weight to the attended region, as this yields the optimal prediction of observed pupil size change. For two examples of the effects of attending either left or right regions on pupil size and weights see Figure 2B,C, respectively. Note that the illustration of Figure 2 used a single observed pupil response as an example. The final model prediction was made on all pupil size changes per condition and per participant instead. In previous studies, we have shown that Open‐DPSM can successfully predict the change in pupil size with an explained variance of 33% (Cai et al. 2024) and that the predicted regional weights can index visual sensitivity in simulated visual field defects (Cai et al. 2025).

Hence, a stronger regional weight indicated an enhanced effect of luminance on pupil response in that region. For instance, central or upper regions were anticipated to have higher weights due to visual field anisotropies, with central/upper regions affecting the pupil more than peripheral/lower regions (Kardon et al. 1991; Naber et al. 2013; Portengen et al. 2021; Strauch et al. 2022a). We found clear corresponding differences in regional weights, thus validating the model further (see details in Data S1). Besides such known anisotropies and, more importantly to the present manuscript, we expected higher regional weights for the attended side because of stronger effects of visual events on the pupil for attended regions.

For more detailed information on Open‐DPSM, see Cai et al. (2024) and Data S1.

#### 2.4.3 Measuring the Location of Covert Attention With Regional Weights

We expected the location of covert attention to be influenced by (1) stimulus‐driven, bottom‐up effects; (2) goal‐driven, top‐down effects; and (3) inherent horizontal imbalances in spatial attention (“pseudoneglect,” usually slightly leftward). Accordingly, we expected higher relative contributions to the pupil response, modeled as regional weights with Open‐DPSM, for (1) regions with more or stronger luminance events, (2) the to‐be‐attended side, and (3) the left over the right side. Furthermore, as a manipulation check for the deployment of top‐down attention, we expected participants to be better at detecting stops on the attended side compared to the unattended side, as covert attention enhances detection, including in‐motion perception (Carrasco 2011; Vater 2019). In addition to task performance, we expected stronger event‐evoked pupillary responses to the stops on the attended side than the unattended side, as stops on the attended side are task‐relevant, and effort‐linked pupil dilation has previously been shown to be stronger for task‐relevant than for irrelevant events (de Gee et al. 2014; Strauch et al. 2020). Thus, we expected stronger pupil dilation to the stops on the attended side than on the other side.

Throughout preprocessing, gaze position was controlled by making the model gaze‐contingent to ensure that potential differences in regional weights are nonartifactual. A linear multiple regression model was used to jointly assess the three effects of the different factors predicting the location of covert attention on the regional weights.

Statistical tests were performed with Python (3.12.3) scipy package (1.13.1) and R (4.1.2) package stats (4.1.2). Pearson's correlations were performed with the “*pearsonr*” function; one‐sample t‐tests and paired‐sample *t*‐tests were performed with “*ttest*_1*samp*” and “*test*_*rel*” in scipy. Multiple regression and model selection were performed with the “*lm*” function in stats.

## 3 Results

### 3.1 Model Performance

Before evaluating whether the regional weights can index covert attention, we first confirmed that pupil size changes to luminance changes could be successfully predicted. The model performance was assessed with the explained variance *R* ^*2*^ and root‐mean‐square error (RMSE) between predicted and observed pupil size changes (see Table 1). Note that while good model performance is a prerequisite for meaningful regional weight estimation, the ability of regional weights to index covert attention cannot be inferred directly from model performance. Whether the estimated regional weights can index covert attention was further evaluated subsequently, addressing bottom‐up, top‐down, and pseudoneglect effects of attention, respectively.

**TABLE 1:** Model performance for different conditions.

Condition	*R* ^*2*^	RMSE
Both	0.48	0.71
None	0.45	0.73
Left	0.49	0.70
Right	0.49	0.70

### 3.2 Bottom‐Up Effects on Regional Weights as an Index of Covert Attention

To determine which side attracted more attention, we extracted luminance changes above ± 5 cd/m^2^ in each region while removing smaller ones as negligible.^1^ The number and amplitude of luminance changes were then taken as a proxy for saliency. The side with more or stronger luminance changes should draw attention in a bottom‐up manner.

Differences in both number and amplitude of luminance changes between sides and differences in regional weights between sides were first calculated by subtracting left from right for both variables, representing the saliency differences between the left and right sides.

Although all participants watched the same movie, individual variations in luminance changes between sides persisted. This variation can be explained by the following factors: (1) Half of the participants viewed horizontally mirrored movies; (2) Trials excluded during preprocessing were different across participants; (3) To account for occasional saccades and small deviations of gaze positions (also see Figure 6), luminance changes were extracted gaze‐contingently. In other words, luminance changes were determined relative to participants' gaze positions over time, rather than relative to the screen or movie center, resulting in individual differences in luminance changes on either side.

**FIGURE 3:**
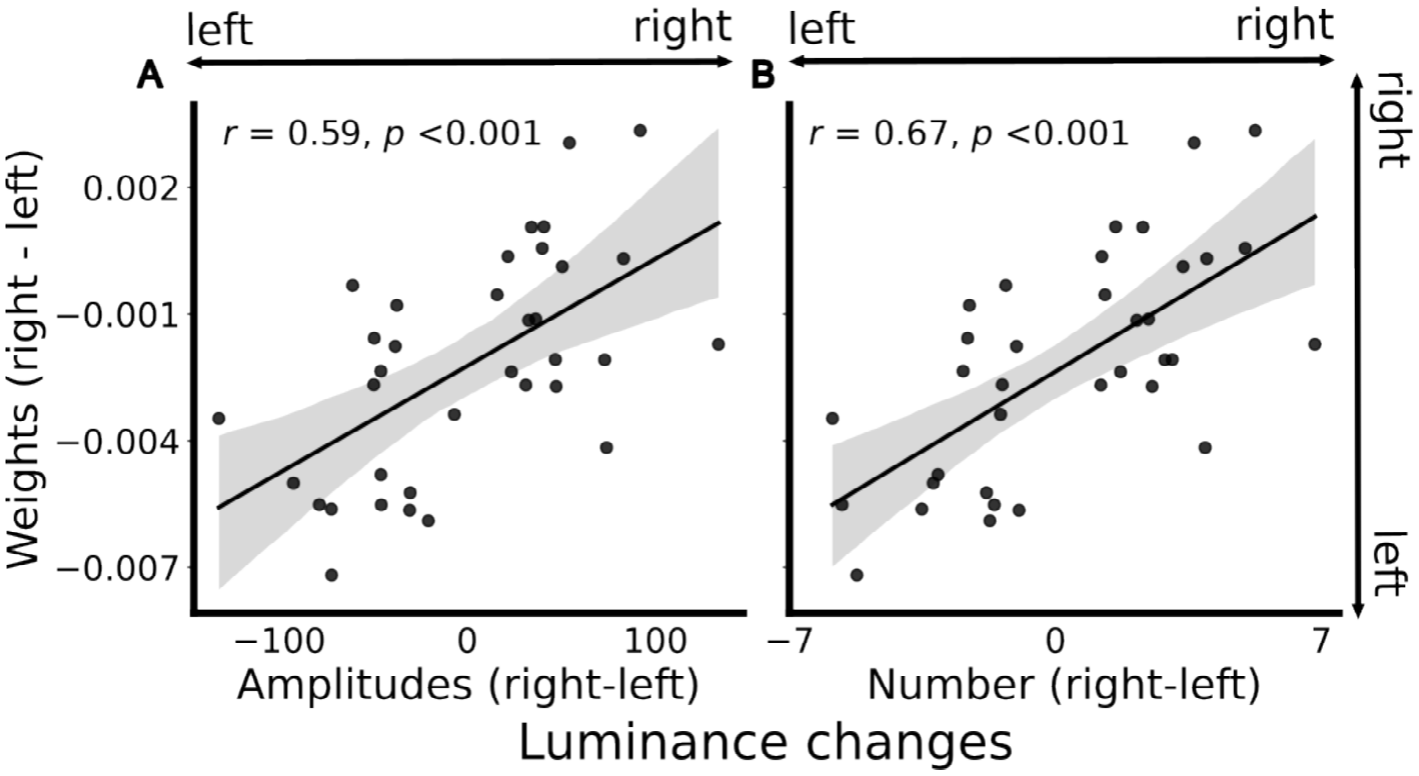
Bottom‐up effects on regional weights as an index of covert attention. (A) higher weight is given to pupil responses on the side with a higher amplitude of luminance changes. (B) Higher weight is given on the side with a higher number of luminance changes. Both relationships are consistent with what we expected from bottom‐up effects on covert attention. Each dot is a participant; shaded bars represent 95% confidence intervals.

We found a positive correlation between regional weights and luminance changes (amplitude of luminance changes: *r* = 0.59, *p* < 0.001; number of luminance changes: *r* = 0.67, *p* < 0.001; Figure 3), indicating that the more salient side affected pupil responses more strongly. This congruent relationship between presumed stimulus‐driven shifts in covert attention and the strength of regional weights implies that the regional weights indeed reflect covert attention.

### 3.3 Top‐Down Effects on Regional Weights as an Index of Covert Attention

**FIGURE 4:**
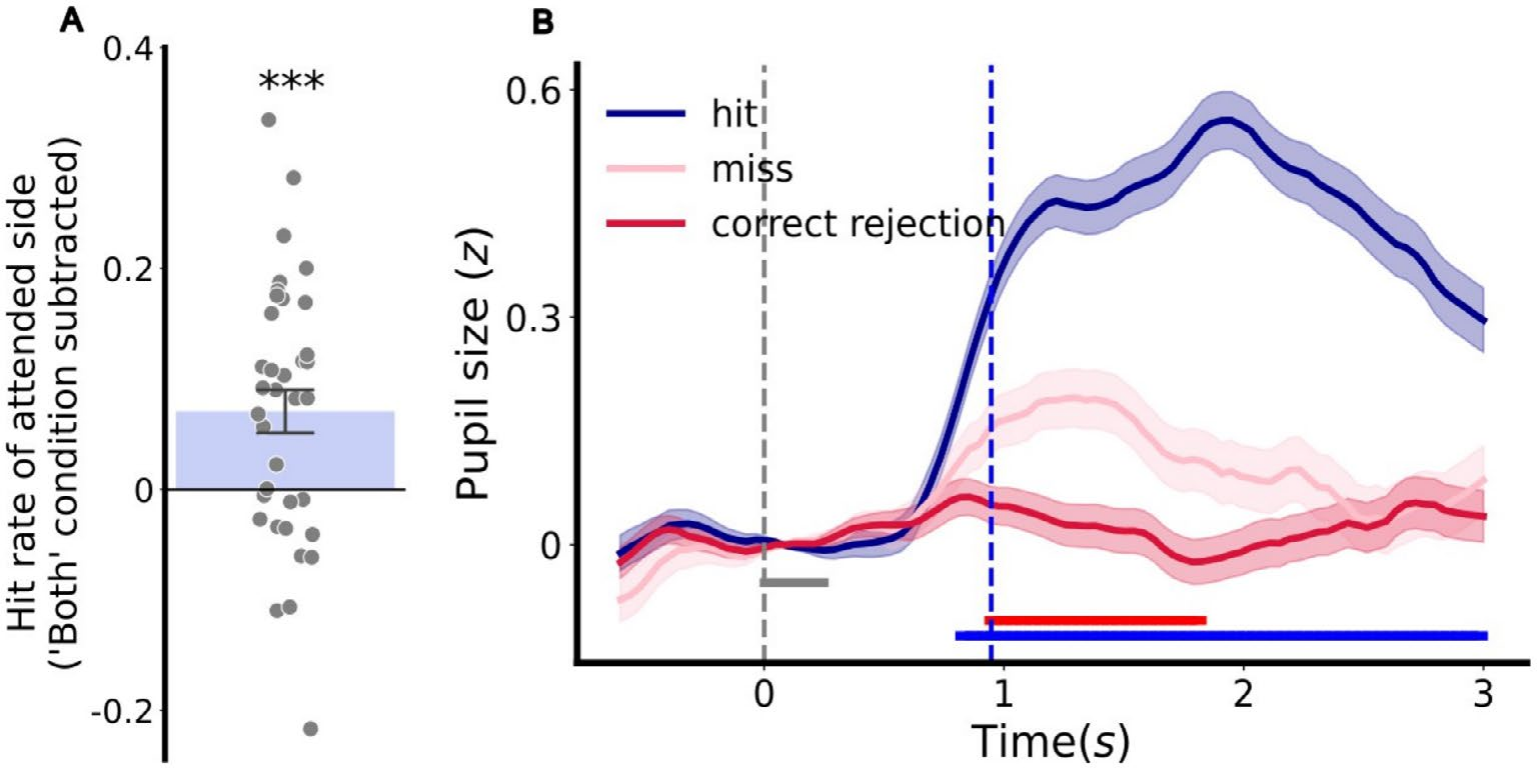
Top‐down covert attention shift. (A) Hit rates of the attended sides in “left” and “right” conditions, with hit rates of the respective sides in “both” condition removed as baseline. Each dot is one participant. (B) Stimulus‐locked pupil dilation averaged for all trials pooled across all participants. Shaded error bands denote the standard error across all trials. Horizontal lines indicate paired *t*‐test *p* < 0.05: blue line: “hit” versus “miss”; red line: “miss” versus “correct rejection”; dashed gray line: stop onset; dashed blue line: average response time (0.95 s); gray line: period used for baseline correction; ***p < 0.001.

We first checked whether the top‐down manipulation of covert attention by conditions was successful. As covert attention in the “both” condition was distributed to both sides, we expected hit rates on either side to be lower than those on the attended sides for the “left” and “right” conditions. The results showed that the attended side in the “left” and “right” conditions was indeed associated with higher hit rates than the same sides in the “both” condition: (1) left sides: “left” condition: *M* = 0.60, SD = 0.13; “both” condition: *M* = 0.54, SD = 0.16; (2) right sides: “right” condition: *M* = 0.64, SD = 0.14; “both” condition: *M* = 0.56, SD = 0.17. A one‐sample *t*‐test showed that the to‐be‐attended side, averaged for the “left” and “right” conditions, had higher hit rates than the respective side in the “both” condition (*t*(35) = 3.61, *p* < 0.001) (Figure 4A).

In addition to hit rates, if attentional instructions were effective, the occurrences of stops on the attended sides should become task‐relevant cognitive events and should elicit an effort‐linked pupil dilation (see de Gee et al. (2014); Strauch et al. (2020)), whereas the stops on the unattended side should induce a much weaker dilation, if any.

Therefore, in the “left” and “right” conditions, we expected a stronger dilation in response to the stops on the attended sides than those on the unattended sides. To examine the effects of higher‐order cognitive events on pupil size, we first subtracted pupil size as predicted by the luminance changes from the overall pupil time series data to reduce the pupil response induced by low‐level features. The pupil time series were then segmented into 3‐s intervals, locked to stop occurrences to obtain event‐related pupillary responses. These event‐related traces were then baseline corrected by subtracting the average pupil size from 0 to 250 ms post stop, where the stop did not yet affect the pupil due to the pupil's sluggish latency. We next standardized each trace by dividing by the standard deviation of all traces. Average traces were calculated for “hits,” “misses,” and “correct rejections” (not for “false alarms” as there was no corresponding stop). “Hits” and “misses” were on the attended side, while “correct rejections” were on the unattended side. As an extra motor response (key press) was presented in all “hits,” which elicits extra pupil dilation (Bumke 1911; Richer and Beatty 1985), pupil dilation in “hits” was incomparable with that in “misses” and “correct rejections.” Although “misses” represented unsuccessful responses to the stimuli (stops of the grating), “misses” should still be associated with (sub‐decision‐threshold) evidence accumulation. Evidence accumulation, in turn, is described as closely linked with pupil dilation (de Gee et al. 2014). Stimuli that are completely unattended and irrelevant (on the unattended side) should therefore not be associated with any accumulation of evidence. To test whether the pupil dilated more for attended stimuli than unattended ones, we compared pupil dilation in “misses” (attended side) to “correct rejections” (unattended side). *T*‐tests over time showed stronger pupil dilation for missed stimuli on the to‐be‐attended side between 0.98 and 1.8 s post‐stimulus onset compared to unattended stimuli (*p* < 0.05, Figure 4B), validating the top‐down manipulation of attention, as stimuli on the to‐be‐attended side indeed received more attention than stimuli presented on the irrelevant side. In summary, both hit rate and pupil dilation to stops indicate that participants adhered to the instruction and attended to the designated side.

**FIGURE 5:**
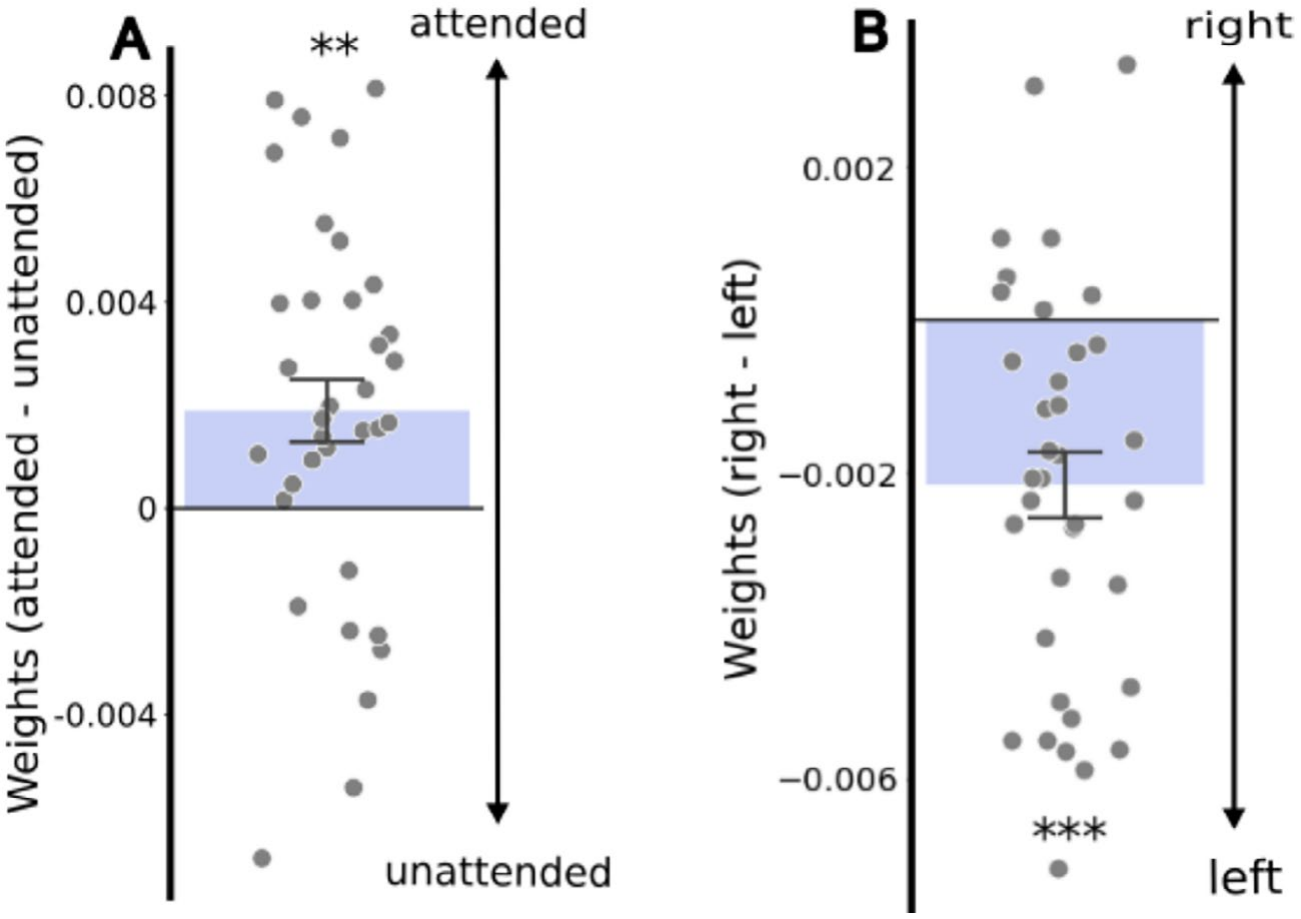
Effects of top‐down shifts and pseudoneglect on covert attention. (A) Difference in regional weights between to‐be‐attended and unattended sides in the “left” and “right” conditions. **p < 0.01; (B) differences in regional weights between right and left sides as an index for pseudoneglect. Each dot is one participant. ***p < 0.001.

Regional weights were higher for the to‐be‐attended than the unattended side for “left” and “right” conditions (*M* = 0.002, SD = 0.004, *t*(35) = 3.12 *p* = 0.004, *d* = 0.53; Figure 5A), indicating that visual events in the attended side contributed more to pupil size change than those on the unattended side. This result illustrates that regional weights were affected accordingly by the attention instruction, implying that the regional weights can reveal a top‐down effect on covert attention. This effect was smaller than in previous simplistic studies (e.g. *d* > 1 in Binda et al. (2013a)), which is not unexpected since movies are highly complex stimuli, introducing greater variability and noise into the data.

### 3.4 Pseudoneglect Effects on Regional Weights as an Index of Covert Attention

Inherent lateral covert attention biases (pseudoneglect) were estimated by subtracting weights between the right and left sides, showing a leftward bias (*M* = −0.002, SD = 0.003, one‐sample *t*‐test: *t*(35) = −5.00, *p* < 0.001, *d* = 0.83; Figure 5C). This leftward bias is consistent with literature on pseudoneglect, showing generally leftward biased attention in young adults as tested here (Jewell and McCourt 2000; Strauch et al. 2022a). This finding is again consistent with our regional weights measuring covert attention, at a higher effect size than that of the previous simplistic pupillometry study (*d* = 0.40 in Strauch et al. (2022a) and *d* = 0.31 in Burns and McIntosh (2023)).

### 3.5 Differences in Gaze Position Do Not Explain Differences in Regional Weights

**FIGURE 6:**
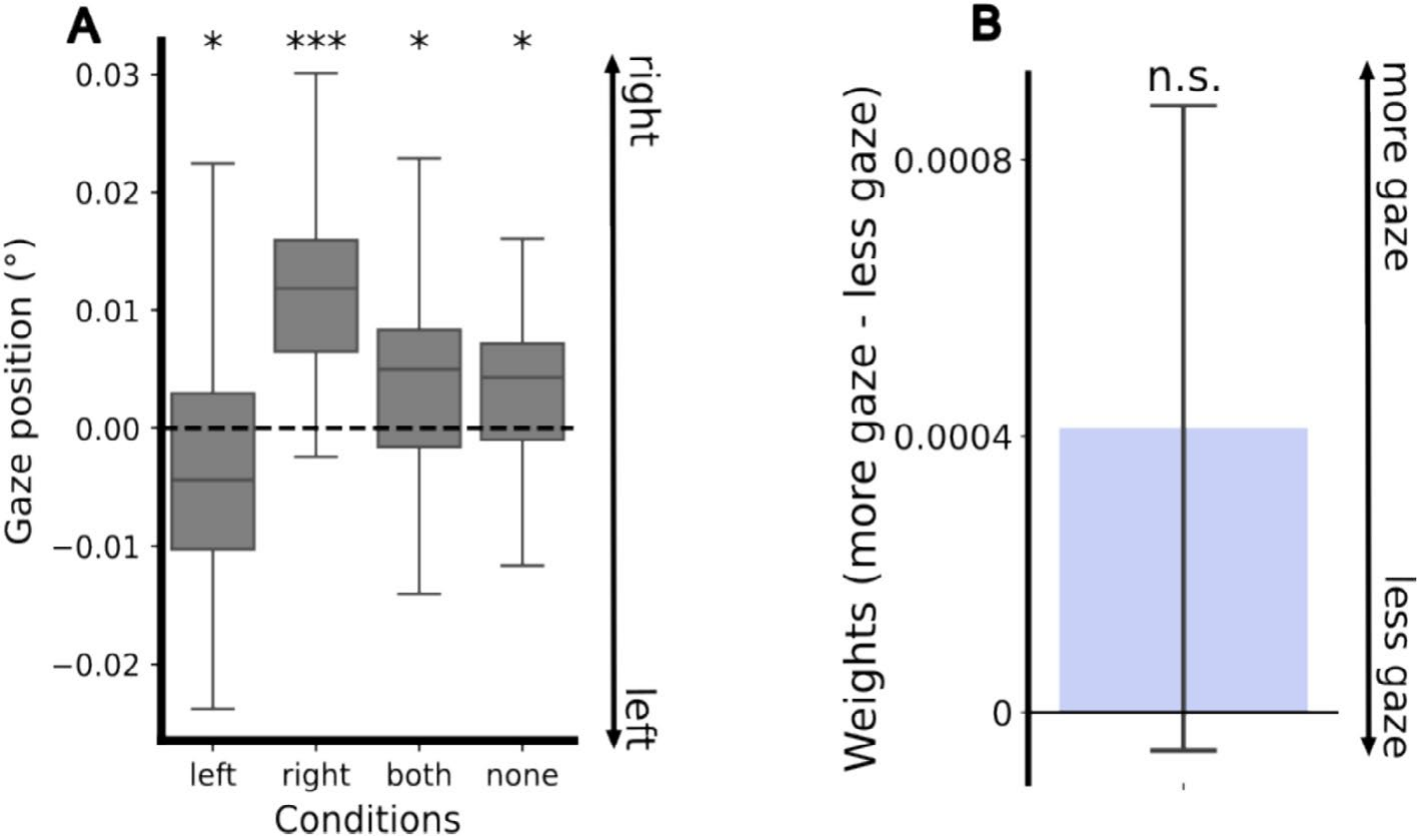
Control analysis on gaze biases. (A) Averaged horizontal gaze positions per participant across conditions. Dashed black line denotes the horizontal midpoint of the movie (0°). Larger value means a more rightward gaze shift; one‐sample *t*‐test against midpoint: *p < 0.05, ***p < 0.001; (B) differences in regional weights between the sides with more gaze and with less gaze.

One may wonder whether the described effects of covert attention on pupil responses resulted from shifted gaze position. Indeed, gaze position was biased towards the attended side in attend left/right conditions (Left: *t*(35) = −2.27, *p* = 0.03, Right: *t*(35) = 8.37, *p* < 0.001), but towards the right in the other two conditions (Both: *t*(35) = 2.57, *p* = 0.01, None: *t*(35) = 2.36, *p* = 0.02; Figure 6A). To ensure that horizontal gaze differences did not cause the differences in weights to pupil size change, pupil size was modeled gaze‐contingently to obtain weights. In other words, the extracted weights were relative to gaze positions over time, rather than to the center of the screen/movie (Figure 1B). Hence, the effects of covert attention are completely independent of those of overt attention. To further assess potential confounding effects of gaze, we compared the weights for the side that received more and less gaze, respectively. Gaze positions per condition per participant were averaged to determine the side with more/less gaze for each pair of weights (left and right sides). A one‐sample t‐test revealed no difference in weights between the more gazed‐at and less gazed‐at sides (*t*(143) = 0.88, *p* = 0.38, Figure 6B). Furthermore, there was no significant correlation between differences in weights between the right and left sides and horizontal gaze positions (*r* = −0.13, *p* = 0.11). Together, this rules out gaze position as a confounding factor.

### 3.6 Evidence for Three Distinct Drivers of Covert Attention

A multiple regression model was used to assess the effects of the three different drivers of covert attention (bottom‐up, top‐down, and pseudoneglect) on regional weights, allowing us to study possible interactions between those effects. This model predicted differences in regional weights between the right and left sides using all three drivers of covert attention and gaze position as a control variable. For bottom‐up effects, as the number and amplitude of luminance changes were highly correlated, they were averaged into a single variable to avoid multicollinearity. The optimal model was determined using AIC and BIC backward selection starting from the full model. Models selected by AIC and BIC were different. AIC backward selection led to the inclusion of intercept (pseudoneglect), the main effects of attention instruction and luminance changes, and BIC backward selection led to the inclusion of only intercept and luminance changes. Neither model selected by AIC nor BIC included gaze position or any interactions. As our principal purpose was to test all three effects (instead of finding the parsimonious model), we followed AIC‐based selection:

$$\begin{array}{c} \text{regional weights}\;\left(\text{right}–\text{left}\right)=\beta_{0}\;\left(\text{pseudoneglect}\right)\\ +\beta_{1}\times {\text{attention instruction}}_{\text{right}}\\ +\beta_{2}\times {\text{attention instruction}}_{\text{none}}\\ +\beta_{3}\times {\text{attention instruction}}_{\text{both}}\\ +\beta_{4}\times \text{luminance changes}\;\left(\text{right}–\text{left}\right)+\epsilon \end{array}$$

The model was statistically significant (*F* (4, 139) = 4.69, *p* = 0.001, *R* ^2^ = 0.09). For the bottom‐up effect, weights were stronger with higher luminance change amplitudes/number (*β*_4_ = 0.22, *p* = 0.009). For the top‐down effect, regional weights were stronger for right side in right, both and none conditions than left condition (Right condition: *β*_1_ = 0.72, *p* = 0.002; None condition: *β*_2_ = 0.48, *p* = 0.03; Both condition: *β*_3_ = 0.58, *p* = 0.01, left condition was used as baseline). For pseudoneglect, a significant negative intercept indicated higher weights for the left side (*β*_0_ = −0.44, *p* = 0.005). The regression result demonstrated the unique contributions of the three covert attention effects, after accounting for their influences on each other. Note that ignoring model selection and including all possible interactions yielded clearly non‐significant interactions (all *p* > 0.57).

## 4 Discussion

We here tested a pupillometry‐based physiological index of the locus of covert attention using dynamic and complex stimuli. We modeled the relative contribution of luminance changes in different locations across the visual field to pupil responses (i.e. regional weights). The spatial distribution of regional weights served as our index of covert attention. We showed that the regional weights were indeed stronger in the parts of the visual field that were plausibly attended more strongly. Specifically, we identified three independent drivers of covert attention as indexed by regional weights: (1) a bottom‐up effect: regional weights were stronger on the side of the movie with more and stronger visual events, consistent with salient stimuli capturing more attention; (2) a top‐down effect: regional weights were stronger for to‐be‐attended sides, consistent with the instructed attentional shift; and (3) a pseudoneglect effect: regional weights were overall stronger on the left side, in line with the small, inherent leftward predisposition of spatial attention. Previous studies have illustrated that the PLR is a physiological index of covert attention, separately for top‐down attention (Binda et al. 2013a; Naber et al. 2013), bottom‐up attention (Mathôt et al. 2014), and inherent lateral biases of attention (Strauch et al. 2022a; Ten Brink et al. 2023). The current study is the first to provide evidence that the PLR can concurrently index all three drivers of covert attention shifts in dynamic environments, which further enables the exploration of whether different drivers of covert attention modulate each other.

Firstly, the bottom‐up capture of attention by salient events has been firmly established (for reviews, see Burnham (2007); Itti and Koch (2000); Theeuwes (2010, 2019)). Using the number and amplitude of luminance changes as an approximation of saliency, we showed that regional weights were higher for the more salient side of the movie, revealing bottom‐up attentional capture (Figure 3). Secondly, the top‐down effects of covert attention (Müller and Rabbitt 1989; Posner et al. 1980; von Helmholtz 1867; Yantis and Jonides 1990) were introduced by asking the participants to voluntarily shift their attention according to the instruction. We first verified the successful manipulation of attention by the improved performance in stimuli detection under conditions where attention was directed to a single side, compared to the condition that required dividing attention between both sides. This indicates that covert attention was shifted as instructed. To complement behavioral performance, we also showed that the stimuli instructed to be attended evoked larger effort‐linked pupil dilation than the irrelevant stimuli on the unattended side (Figure 4). As task‐relevant stimuli evoke greater pupil dilation than irrelevant stimuli (de Gee et al. 2014; Strauch et al. 2020), this effect provides convergent physiological evidence that top‐down attention was shifted as instructed. Consistent with the instructed attention shifts, regional weights were also higher for the to‐be‐attended side according to instruction (Figure 5). It is worth noticing that in the current study, attention was explicitly manipulated across different conditions to demonstrate a top‐down effect of covert attention on the PLR. Future studies are necessary to assess whether greater regional weights can be linked directly to better behavioral performance, without explicit manipulation of attention. Thirdly, the effect of pseudoneglect, an inherent leftward attention bias, has been found in many studies using behavioral (for reviews, see Friedrich et al. (2018); Jewell and McCourt (2000)) and recently also pupillary measurements (Strauch et al. (2022a); Ten Brink et al. (2023); see also Burns and McIntosh (2023)). Similarly, regional weights were overall higher on the left side, irrespective of bottom‐up and top‐down attentional effects.

While both top‐down and bottom‐up attention improve behavioral performance, previous studies have revealed key differences, such as their effects on spatial resolution and second‐order texture contrasts (Barbot et al. 2012; Carrasco and Yeshurun 1998; Yeshurun et al. 2008), which indicate that these two drivers of covert attention may reflect independent mechanisms competing for where attention should be directed (Müller and Rabbitt 1989). It is not fully clear whether bottom‐up attention is modulated by top‐down attention in behavioral tasks, that is, whether the bottom‐up effect of salient stimuli would be attenuated or even completely suppressed by top‐down attention (Berger et al. 2005; Berlucchi et al. 2000; Chica et al. 2006, 2013; Chica and Lupiáñez 2009; Folk et al. 1992; Jones and Forster 2013; Müller and Rabbitt 1989; Pinto et al. 2013; Riggio and Kirsner 1997; Theeuwes 1991; Van der Lubbe et al. 2005; Yantis and Jonides 1990). The question on (in)dependence of these different effects on attention is also debated in clinical research, that is, whether patients with hemispatial neglect exhibit impairments in both top‐down and bottom‐up attention, or only one of them (Bartolomeo et al. 2001; Bartolomeo and Chokron 2002; Vuilleumier and Schwartz 2001). When predicting the regional weights as an index of covert attention from bottom‐up, top‐down, and pseudoneglect effects, we found independent, non‐interacting contributions of top‐down and bottom‐up spatial attention. This means that saliency effects were similar across all top‐down attention conditions. This may be surprising given previous literature demonstrating the dominance of top‐down attention (e.g. the classic Yarbus (1967) study). Here, however, it is apparently not possible to fully ignore the visual events in the movie, even though those visual changes were completely irrelevant to the task. Hence, our physiological approach to measuring covert attention behavior‐free suggests that top‐down and bottom‐up attention indeed act independently (in line with Berger et al. (2005); Berlucchi et al. (2000); Chica et al. (2006); Jones and Forster (2013); Pinto et al. (2013); Riggio and Kirsner (1997)). Additionally, pseudoneglect is known to be subtle in effect size (Jewell and McCourt 2000), so one may wonder whether its effects persist when stronger effects of covert attention are present (e.g. top‐down attentional manipulation). Our findings suggest that pseudoneglect persisted also when both top‐down and bottom‐up effects affected covert attention. This is in line with recent evidence which showed that participants tend to start searching on the left side of the screen even if they know that a target is on the right (Nuthmann and Clark 2023). To date, no study has shown the relationship across all these drivers of covert attention simultaneously and physiologically. Our results suggest that in environments as complex as in the present study, all drivers jointly and additively rather than interactively drive covert attention.

Moreover, inferring covert attention with pupil responses was restricted to simplistic visual stimulation in rigorously controlled environments in previous studies. For complex stimulus materials, researchers had to rely on overt responses to infer covert attention. Reluctance to use the PLR in complex environments stems from difficulties in disentangling pupillary responses to low‐level features and higher‐order factors (see Mathôt (2018); Strauch et al. (2022b) for reviews). Hence, it is generally considered essential to maintain identical or similar luminance across all stimuli to study cognition with pupillometry (Aktar et al. 2021; Bradley and Lang 2015; Juvrud et al. 2022; Mitre‐Hernandez et al. 2021), which largely limits the applicability and flexibility of applying it to real‐world scenarios. We here demonstrated that the PLR is capable of indexing covert attention even within highly dynamic and complex stimuli. Our approach, therefore, opens up possibilities to study covert attention unobtrusively, without the need for overt responses, and with complex stimulus material.

### 4.1 Applications

Tracking covert attention is challenging, as it cannot be observed directly with gaze. Behavioral responses can be used to measure covert attention manually, but they may introduce additional interference to the main task and even bias covert attention itself. As an implicit measurement, the PLR has provided valuable insights on covert attention across several tasks and fields, such as spatial‐numerical associations (Salvaggio et al. 2022), visual working memory maintenance (Unsworth and Robison 2018), or breadth (Brocher et al. 2018; Tkacz‐Domb and Yeshurun 2018) and spatiotemporal dynamics of attention (Naber et al. n.d.). Now, this technique can be used also in applied research where it is particularly relevant, such as in driving, professional sports, and gaming, or for research into attention deficiency. Hereby, participants can be kept naive to the study's purposes, which allows for a more objective and convenient assessment.

For instance, in the aforementioned example of driving, previous studies captured or assessed covert attention by superimposing additional stimuli or tasks on the driving task (Costa et al. 2018; Crundall et al. 2002; Tuhkanen et al. 2019), which interfered with the primary task itself and may bias the measurements. The current study may provide a possibility to study covert attention maintenance unobtrusively in such dynamic contexts. Another application lies in studying clinical alterations of covert attention, such as in hemispatial neglect after brain damage. Conventional behavioral tests are often cognitively demanding (Bolognini and Vallar 2020; Ten Brink et al. 2023) and our here‐introduced method may provide a more feasible alternative.

### 4.2 Future Directions

The current modeling method requires a substantial amount of data to estimate regional weights, which, in turn, represent the distribution of covert attention. As each condition contained data from 8 min of movie‐watching, and 70% of the data were used for model training, around 5–6 min of movie‐watching data were required. Consequently, we can only infer an overall allocation of covert attention for this period. Future studies could focus on improving the estimation of regional weights by optimizing stimuli and the model. This should increase the temporal resolution of our covert attention tracker. Similarly, as we here only tested covert attention to the left and right sides, respectively, the spatial resolution of the approach is currently still constrained. Nevertheless, the model independently estimated the weights for more than 40 regions (Figure 1B), representing the potential to enhance spatial resolution for the current method. The efficacy and reliability of the model in targeting more and smaller regions, however, remain to be tested. We expect that this method has the potential to give a much better spatially resolved estimation of covert attention and may eventually allow maps of covert attention, but this may only be achievable with sufficient data. Hence, future studies should investigate the trade‐offs between the temporal and spatial dimensions of the method to determine the optimal amount of data for precise indices of covert attention.

All three here investigated drivers of covert attention significantly (and independently) predicted differences in regional weights. We reached our goal of testing and validating the link between covert attention and regional weights, but substantial variation was left unexplained. Part of this unexplained variance is caused by random noise in the estimated regional weights, but much room exists for improving the current approach further by incorporating other drivers of covert attention, and thus gaining an even more sensitive measure. As one example, considering the complexity of movies, it is possible that bottom‐up capture (saliency) of attention was not measured to its full extent. For instance, the distribution of saliency across the movie clips' locations was estimated merely based on the strength and frequency of luminance changes. Many other factors, such as color, spatial frequency, presence of faces, and motion, can contribute to saliency (Evangelopoulos et al. 2013; Itti et al. 1998; Kucerova and Sikudova 2011; Rogalska and Napieralski 2018; Xu et al. 2015; Yildirim and Süsstrunk 2015). Consequently, we expect that more advanced saliency models should predict regional weights even more strongly.

As previously mentioned, visual events were extracted gaze‐contingently, which means that the regional weights, and thus, the measured covert attention were anchored to the gaze position. Hence, we argue that although the current study required a central fixation throughout the experiment, this is not essential. Eliminating this restriction would facilitate additional applications, such as studying the link between covert and overt attention in free viewing. Nevertheless, it is worth noticing that with our restricted fixation position, the model could account for 47% of the variance in pupil size changes (averaged across all four conditions), exceeding the performance observed in our earlier study under free‐viewing conditions (33%, see Cai et al. 2024). The discrepancy in the performance of the model can be attributed to the unexplained pupil size changes caused by eye movements (Binda and Morrone 2018; Koevoet et al. 2023, 2024) and pupil foreshortening errors (Hayes and Petrov 2016; Mathôt and Vilotijević 2023) in free viewing. There are various possible ways to further optimize model performance (see Cai et al. (2024) for suggestions on improving Open‐DPSM) and thereby the use of it to infer covert attention. For instance, the current model only accounted for luminance changes while other low‐level features evoking pupil responses, such as changes in color, spatial frequency, and orientation (Barbur et al. 1992; Hu et al. 2019; Kimura and Young 1995) were not considered. These steps should facilitate improving both the spatial and temporal resolution of covert‐attention tracking.

### 4.3 A Tutorial on How to Index Covert Attention With Dynamic Stimuli Using Open‐DPSM

The Open‐DPSM toolbox is available at https://github.com/caiyuqing/Open‐DPSM. We recommend users to first become familiar with our modeling approach by using our interactive illustration (“Regional weights illustration” folder). The modeling procedure mainly involves: (1) How luminance changes are extracted in each region; (2) How pupil responses are modeled with convolution (multiplication of a so‐called response function, a mathematical approximation of the pupil response with the extracted luminance changes) for each region; (3) How regional weights are estimated by incorporating differentiated contributions of luminance changes in different regions as so‐called regional weights (For instance, luminance changes in the center contribute more to pupil responses than in the periphery and thus regional weights in the center are expected to be estimated higher than in the periphery). The best combination of regional weights is determined by model optimization.

In practice, researchers who are interested in studying covert attention using pupil size changes in complex environments can use our Python script or our graphical user interface of the toolbox (in “v2” folder). Simply load the video file of the presented screen content and a corresponding eye‐tracking file. The toolbox will output the estimated regional weights, as well as the overall model fit, describing how well the model captured pupil size changes. Then regional weights across regions and conditions can be compared to answer the designated questions. More detailed guidelines for using the toolbox are provided on the GitHub page.

## 5 Conclusion

The current study illustrated that the pupillary light response (PLR) can be used as a sensitive physiological marker of covert attention, also with complex stimuli when modeled appropriately. With Open‐DPSM, we demonstrated that luminance changes in regions that captured more attention contributed more to the PLR. This study is the first to assess the multiple effects of covert attention (i.e. exogenous, endogenous, pseudoneglect effects) on pupil responses simultaneously and beyond simplistic and controlled stimuli. We see potential for this technique in studying covert attention, especially in use cases where overt responses are undesirable or stimuli are complex—reflecting most real‐world scenarios.

## Author Contributions

**Yuqing Cai:** conceptualization, data curation, formal analysis, methodology, project administration, software, visualization, writing – original draft, writing – review and editing. **Stefan Van der Stigchel:** funding acquisition, supervision, writing – review and editing. **Julia Ganama:** conceptualization, data curation, writing – review and editing. **Marnix Naber:** conceptualization, project administration, supervision, writing – review and editing. **Christoph Strauch:** conceptualization, funding acquisition, project administration, resources, supervision, writing – review and editing.

## Disclosure

The experiment was not preregistered.

## Ethics Statement

The data collection was approved by the local ethics board of the Faculty of Social Sciences of Utrecht University upfront (22‐530).

## Consent

All participants gave informed consent before the start of the experiment.

## Conflicts of Interest

The authors declare no conflicts of interest.

## Supporting information


Data S1.

## Data Availability

All data (eye tracking and behavioral responses) are available via https://osf.io/m5h2f/. The code for Open‐DPSM is available at https://github.com/caiyuqing/Open‐DPSM; codes for analyzing behavioral responses and for extracting ERPR can be found via https://osf.io/m5h2f/.

## Endnotes

^1^
Note that this cutoff is arbitrary in its specific value, and cutoffs of ± 3 and ± 7 cd/m^2^ led to similar findings (see Figure S3).
