# Supplementary material for: Uncovering Distinct Drivers of Covert Attention in Complex Environments With Pupillometry
Source: Psychophysiology. 2025 Mar 19;62(3):e70036. doi: 10.1111/psyp.70036 (PMC11920946; doi:10.1111/psyp.70036)
Supplement: Supplementary file 1 — Data S1. [file PSYP-62-e70036-s001.docx]

# Supplementary materials for "Uncovering covert attention in complex environments with pupillometry"

Yuqing Cai1, Stefan Van der Stigchel1, Julia Ganama1, Marnix Naber1, and Christoph Strauch1

1Experimental Psychology, Helmholtz Institute, Utrecht University, The Netherlands

# Supplementary materials for "Uncovering covert attention in complex environments with pupillometry"

# Method

## Visual field regions

To map the contributions of visual field regions to pupil responses (i.e., sensitivities), the visual field was divided into separate regions, and visual events were extracted individually for each region. In Cai et al. [(2023),](#_bookmark3) the regions were rectangular in shape. Here, we adopted the shape of a circular map, which divided the visual field into 44 regions across five eccentricity rings, following the standards in the field of pupil perimetry (Bell et al., 2010; Portengen et al., 2022; Sabeti et al., 2017; Wilhelm et al., 2000). The map covered a 60° horizontal and vertical field of vision, which matched the horizontal visual angle of the movie.

## Gaze-centered visual events extraction

Although the participants were required to fixate at the center cross throughout the experiment, shifts in gaze position were inevitable, which resulted in corresponding shifts in the position of the video image projected onto the retina. For example, if gaze shifted toward the top left of the screen, the center of the retina would not correspond to the center of the screen. As the background surrounding the monitor was black, we embedded the movie frames in a larger black rectangle to simulate what really fell on the retina of the participants. At each time point, the center of the circular regions was re-aligned with the gaze position, which was defined as the center of the visual field. Visual events (i.e. luminance changes and contrast changes) were extracted for each region independently.

Luminance changes in each region were calculated as the difference in luminance, averaged over all pixels in a corresponding region, between consecutive frames. Contrast changes were approximated by the absolute value of the luminance changes.

## Modeling the pupil size change to visual events

We adopted the convolutional model to model the continuous pupil size changes to all luminance and contrast changes and thereby obtain the relative contributions of visual events to pupil size changes in each visual field region, as described before. The convolutional modeling method approximates pupillary responses to visual events with a prototypical pupil response to stimulation, a so-called response function (Supplementary [Figure](#_bookmark0) 1A). Two unified response functions were convolved with luminance changes and contrast changes in all regions respectively (see Supplementary [Figure](#_bookmark0) 1B for this procedure for luminance change as an example). The predicted pupil size change was modeled as the sum of all the responses at each time point (Supplementary [Figure](#_bookmark0) 1C, green line). The shape of the two response functions was controlled by two free parameters respectively, and an additional free parameter was introduced to control for the relative amplitude of the peak for contrast response compared with luminance response. Note that contrast responses produce temporary (transient) responses that go back to baseline within several seconds, while luminance changes elicit sustained responses. Hence, the pupil size change trace modeled for luminance changes (not contrast) was accumulated to produce sustained responses (Supplementary [Figure](#_bookmark0) 1E, black line). To discern the relative contribution of each region to pupil size changes, 44 additional parameters were incorporated as regional weights. Predicted pupil size changes in each region, induced by convolution of luminance and contrast changes with response functions were then multiplied by the respective regional weight (Supplementary [Figure](#_bookmark0) 1C for an example when one region gets higher weights than the other). The final predicted pupil size changes across all the regions were computed by averaging all these weighted predictions (see Supplementary [Figure](#_bookmark0) 1D an exemplary illustration of the result of combining two regional weights into corresponding modeled pupil size changes). The predicted pupil size changes (red line in Supplementary [Figure](#_bookmark0) 1E, accumulated because it is pupil response for luminance changes) were then compared with the real pupil size changes (black line in Supplementary [Figure](#_bookmark0) 1E). In total, our model encompassed 49 free parameters.

Nelder-Mead simplex search algorithm was adopted to search through different

combinations of parameter values, minimizing the root mean squared error (RMSE) between the predicted and the actual pupil size change. To prevent overfitting, we used

cross-validation, dividing all trials in each participant randomly into training (70%) and testing (30%) datasets. Five iterations of cross-validation were performed, and all reported results were the averaged testing results across iterations. To evaluate the model performance, R-squared and RMSE between the predicted and the observed pupil size changes were calculated for each condition (for the full procedure see Cai et al. [(2023)).](#_bookmark3)

## Regularization on regional weights

Due to the interrelated nature of the content (and thus luminance changes) across (movie image) regions, visual events within different regions exhibited high correlation (especially for those that were adjacent to one another). As an extension to Open-DPSM (Cai et al., [2023),](#_bookmark3) ridge regression, a regularization technique that suppresses artifacts in weights caused by multicollinearity, was therefore applied to bolster the predictive accuracy of the linear regression model and to mitigate overfitting. Ridge regression, instead of other regularization techniques (e.g., Lasso, Elastic Net), was chosen because, in our previous study (Cai et al., 2025), we found that ridge regression performed the best for enhancing the regional weights’ ability in distinguish visual sensitivity of different locations across the visual field. Ridge regularization introduces a penalty term into the cost function, discouraging excessively large coefficients for predictors (i.e., regional weights). Notably, only the many parameters associated with regional weights underwent regularization, while the parameters of the response function remained unchanged. The regularization process was implemented using the "Ridge" class within the "linear" module of the "sklearn" package. The strength of regularization was fine-tuned via the "alpha" parameter. The optimal alpha value was determined by an evaluation of the root mean squared error (RMSE) of testing sets (an alpha resulting in the smaller test RMSE represents a better model fit) and the difference in RMSE between training and testing sets (an alpha resulting in the least difference in RMSE corresponds to a model with minimal overfit).

# Figure 1

*An illustration of the convolutional approach of Open-DPSM*


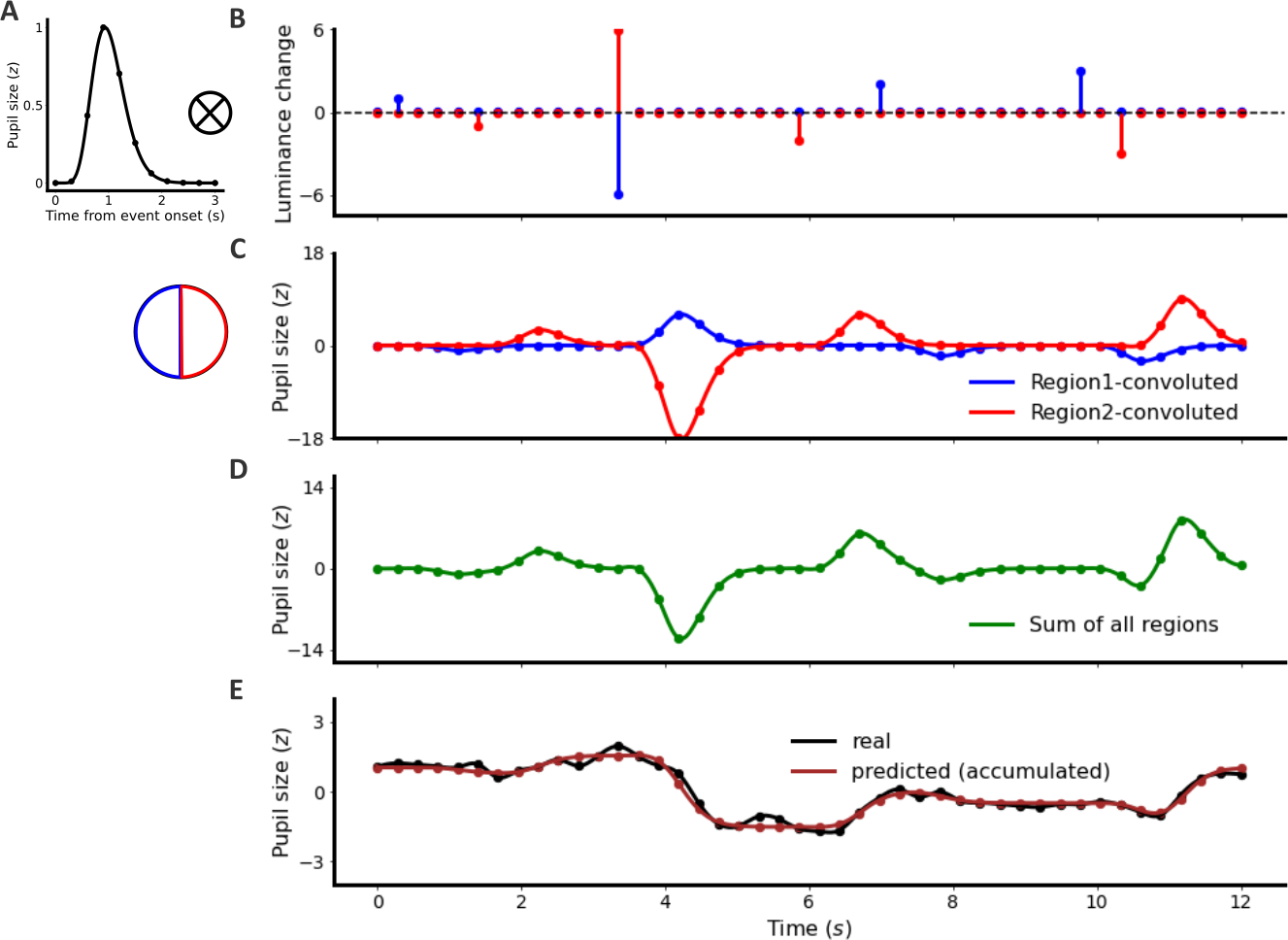


*Note:* ***A*** *Pupil response function used for convolution.* ***B*** *Illustrative data of luminance changes over time in left and right visual field regions (see Fig. 1B in the main text for actual regions), represented by blue and red, respectively. Changes can be either positive (dark to bright) or negative (bright to dark).* ***C*** *Simulated pupil responses by convolving the response function in A with luminance changes in B, showing predictions of transient changes for each of the two regions. Note that pupillary responses are opposite to the luminance changes as luminance increments induce a constriction. In this case, the red region is attended that the "red" luminance changes elicited a stronger pupil response than the "blue" luminance changes.* ***D*** *Overall predicted transient responses (green) calculated by the sum of the two weighted predictions in panel C.* ***E*** *Accumulated (sustained) prediction of pupil response (red), which is the final predicted luminance change by the model, and real pupil size change (black)*

# Supplementary Results

## Visual field anisotropies

Previous studies show that visual events in the central and upper visual field elicit stronger pupil responses than the ones in the peripheral and lower regions (Kardon et al., 1991; Istiqomah et al., 2022; Naber et al., 2013; Portengen et al., 2021; Strauch et al., 2022). If the model adequately models pupil size changes, those two anisotropies should therefore also be reflected with regional weights. The average of regional weights across conditions for each participant was first calculated. Then, we compared the average regional weight in the first three inner rings (within 15.7° of the visual angle on either side) with that of the two outer rings. We found significantly higher weights in the center than in the periphery (t(35) = 15.23, p < 0.001). Similarly, we also found higher weights in upper regions relative to those in bottom regions (t(35) = 10.72, p < 0.001), which aligned with the expected anisotropies of the visual field (see Supplementary Figure 2 for an overall distribution of regional weights averaged across all participants). These results indicate that the regional weights reflect well-established visual field anisotropies on the pupil response, thus further validating the model.

Figure 2

Overall regional weights distribution


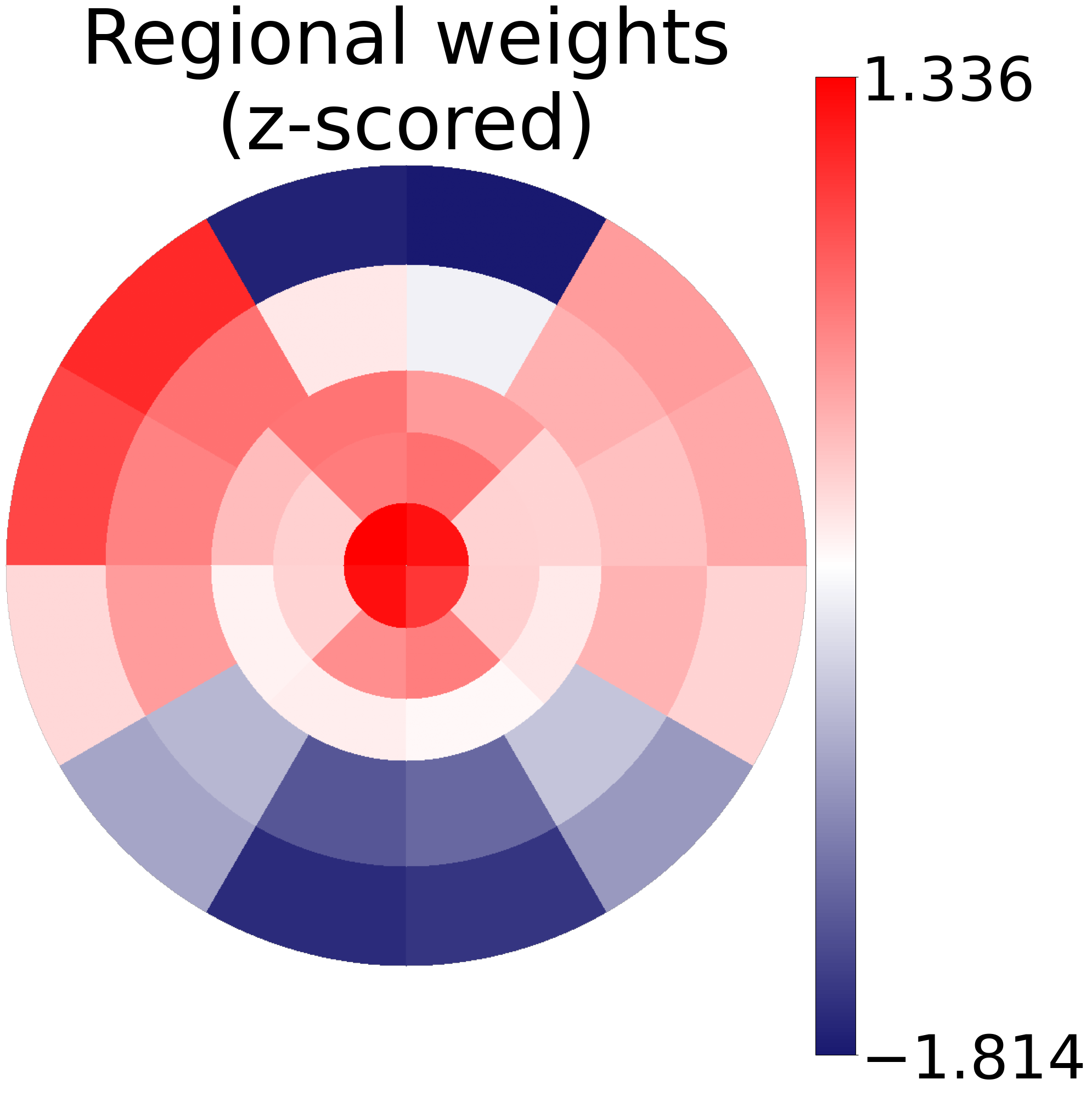


Note: Averaged regional weights across all participants and all conditions.

## Bottom-up effects on the regional weights (other thresholds for removing small luminance changes)

In addition to the threshold of ±5 cd/m² reported in the main text, we tested thresholds of ±3 and ±7 cd/m² to remove small luminance changes. With either threshold, positive correlations between saliency and regional weights were found, demonstrating the expected bottom-up effect: luminance changes in more salient regions contributed more to pupil size changes (see Supplementary Figure 3).

**Figure 3**


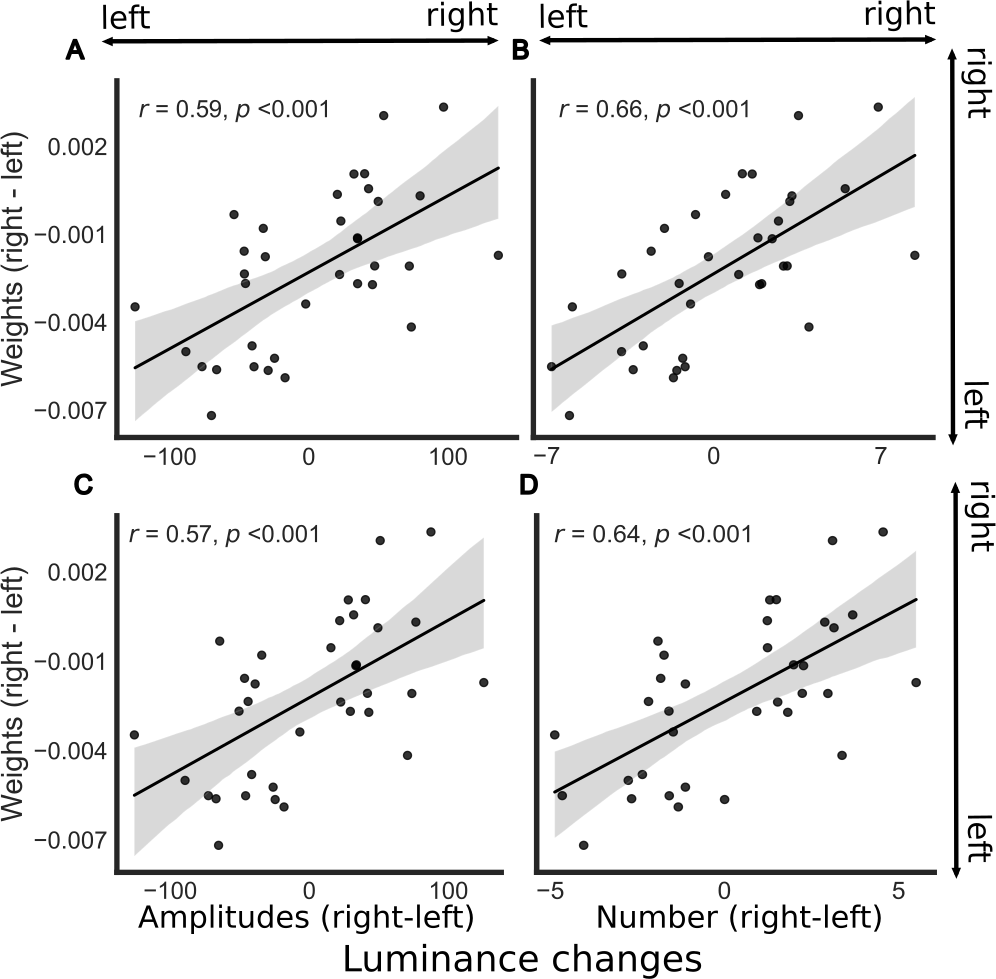
*Bottom-up effects on regional weights as an index of covert attention*

*Note: A and C show that weights are higher on the side with higher amplitude of luminance changes; B and D show weights are higher on the side with higher number of luminance changes. The first row (A and B) and second row (C and D) are the results with thresholds of ±3 and ±7 cd/m2 to remove small luminance changes respectively. All relationships are consistent with bottom-up effects on covert attention observed in the main manuscript with a cutoff of ±5 cd/m2. Each dot is a participant, and shaded bars represent 95% confidence intervals.*

# References

Cai, Y., Strauch, C., Van der Stigchel, S., & Naber, M. (2023). Open-DPSM: An open-source toolkit for modeling pupil size changes to dynamic visual inputs. *Behavior Research Methods*. <https://doi.org/10.3758/s13428-023-02292-1>

Istiqomah, N., Suzuki, Y., Kinzuka, Y., Minami, T., & Nakauchi, S. (2022). Anisotropy in the peripheral visual field based on pupil response to the glare illusion. *Heliyon*,

*8* (6).

Strauch, C., Romein, C., Naber, M., Van der Stigchel, S., & Ten Brink, A. F. (2022). The orienting response drives pseudoneglect—Evidence from an objective pupillometric method. *Cortex*, *151*, 259–271. <https://doi.org/10.1016/j.cortex.2022.03.006>
